# Ship Fever

**Published:** 1848-03

**Authors:** 


### SHIP FEVER.

The ship or typhus fever seems to be prevailing to a great extent in the hospitals of New Orleans, more especially among the emigrants. In all our Atlantic cities, particularly north of the Potomac, we have had, during the winter, more or less of the disease in the receptacles of the poor emigrants, whether public institutions or private habitations. In the latter abodes, the destitution is often so great that one cannot be surprised that disease should reign, but rather that any individuals so exposed should escape.

---

The length of several communications contained in the present number, has prevented the insertion of the usual variety in our *Record,* as well as excluded some shorter articles, and notices of new publications. These shall be attended to in our next.

